# Unsteady Squeezing Flow of Carbon Nanotubes with Convective Boundary Conditions

**DOI:** 10.1371/journal.pone.0152923

**Published:** 2016-05-05

**Authors:** Tasawar Hayat, Khursheed Muhammad, Muhammad Farooq, Ahmad Alsaedi

**Affiliations:** 1 Department of Mathematics, Quaid-I-Azam University, 45320, Islamabad, 44000, Pakistan; 2 Nonlinear Analysis and Applied Mathematics (NAAM) Research Group, Department of Mathematics, Faculty of Science, King Abdulaziz University, P. O. Box 80257, Jeddah, 21589, Saudi Arabia; 3 Department of Mathematics, Riphah International University, Islamabad, 44000, Pakistan; Tsinghua University, CHINA

## Abstract

Unsteady flow of nanofluids squeezed between two parallel plates is discussed in the presence of viscous dissipation. Heat transfer phenomenon is disclosed via convective boundary conditions. Carbon nanotubes (single-wall and multi-wall) are used as nanoparticles which are homogeneously distributed in the base fluid (water). A system of non-linear differential equations for the flow is obtained by utilizing similarity transformations through the conservation laws. Influence of various emerging parameters on the velocity and temperature profiles are sketched graphically and discussed comprehensively. Analyses of skin fraction coefficient and Nusselt number are also elaborated numerically. It is found out that velocity is smaller for squeezing parameter in the case of multi-wall carbon nanotubes when compared with single-wall carbon nanotubes.

## Introduction

Due to the rapid progress in thermal engineered systems and heat exchangers, enhancement of rate of heating or cooling has always been in demands for heating/cooling industrial processes. Poor heat transfer properties of traditional coolants have been an indispensable challenge for the scientists and engineers in heat transfer media and limits their applications. In fact, the working fluids play a major contribution in the cooling systems. However, the conventional heat transfer fluids such as water, oil and ethylene glycol, have relatively low thermal conductivities. In this direction a great interest has been shown by the scientists and engineers in order to improve the thermal properties of these fluids. Recently great interest has been developed to analyze the heat transfer via nanofluid. Nanofluids are actually homogenous mixture of base fluid and nanoparticle with size (10–100 nm) diameter. Nanofluid is considered as a promising way for enhancing the capability of heat transfer in fluids. In fact, the outstanding feature of a nanofluid is its superior thermal conductivity comparing to base fluid. Nanoparticles are made from different materials, such as oxide ceramics (Al_2_o_3_, Cuo), metal nitrides (AlN, SiN), carbide ceramics (SiC, TiC), metals (Cu, Ag, Au), carbons (e.g., diamond, graphite, carbon nanotubes, fullerene) and functionalized nanoparticles. The term Nano was first introduced by Choi [1]. Nowadays carbon nanoparticles are utilized due to their higher thermal conductivity. Rate of heat transfer of nanofluids highly depends upon the shape of nanoparticles. The effect of shape of nanoparticles on the heat transfer and thermodynamics performance are studied by Elias et al. [2] and it is found that cylindrical shaped nanoparticle (nanotubes) have better performance in terms of thermal conductivity, heat transfer coefficients and heat transfer rate. Best performance was found for tubes shaped nanoparticle in comparison to bricks, blades, platelets and spherical shaped nanoparticles respectively. A carbon nanotube is a tube-shaped material made of carbon and having a diameter measuring on the nanometer scale. On the basis of structure, the carbon nanotubes are categorized in single-wall and multi-wall carbon nanotubes (SWCNTs and MWCNTs). Carbon nanotubes have wide range of applications such as conductive plastics, structural composite materials, flat-panel displays, gas storage, antifouling paint, micro and nanoelectronics, radar-absorbing coating, technical textiles, ultra-capacitors, atomic force microscope (AFM) tips, batteries with improved lifetime, biosensors for harmful gases, extra strong fibres etc. CNTs are used in medical devices and biosensors due to their higher chemical compatibility with biomolecules, i.e. proteins and DNA and also for purification of contaminated drinking water [3]. In a sheared fluid the orientation of a rigid carbon nanotube and controlling its orientation is investigated by Dong and Cao [4]. In steady and transient flow the orientation of multi-wall carbon nanotube (MWCNT) is studied by Pujari et al. [5]. Turkyilmazoglu [6] explored the heat transfer characteristics of nanofluids due to a rotating disk. Dong and Cao [7] examined the unidirectional superhigh-speed rotatation of carbon nanotubes in linear shear flow. Nayak et al. [8] studied the mixed convection and entropy generation in Cu-water nanofluids in a heated skewed enclosure. Kherbeet et al. [9] performed an experiment to analyze the flow and heat transfer characteristics of nanofluids over a microscale. Peristaltic transport of water based nanofluids is mathematically modeled and studied by Shehzad et al. [10]. Sheikholeslami et al. [11] explored the characteristics of heat transfer for Fe_3_O_4_-water nanofluids with non-uniform magnetic field and forced convection. Effect of slip and wall properties on the peristaltic motion of nanofluids with Joule heating and magnetic field are analyzed by Hayat et al. [12]. Hayat et al. [13] discussed the characteristics of carbon nanotubes in the flow of water with homogenous-heterogenous reactions.

Squeezing flow between parallel plates has gained a considerable interest by the scientists and engineers due its wide range of applications in industrial and biological processes. Such applications involve polymers processing, compression, injection modeling, lubricant system, transient loading of mechanical components, food processing, cooling water and modeling of synthetics transportation inside living bodies. The characteristics of squeezing flow was initially studied by Stefan [14]. Magnetohydrodynamic squeezed flow of nanofluid over a sensor surface is investigated by Haq et al. [15] Features of unsteady squeezing flow of nanofluids between two parallel plates are investigated by Gupta and Ray [16]. Qayyum et al. [17] analyzed the time dependent squeezing flow of Jeffrey fluid between two parallel disks. Hayat et al. [18] analyzed mixed convection squeezing flow of an incompressible Newtonian fluid between two vertical plates. The features of squeezed nanofluid between two parallel plates are analyzed by Sheikholeslami et al. [19]. Dib et al. [20] presented the squeezing flow of nanofluids analytically.

Disclosing the properties of fluids subject to high rate of cooling or heating the scientists and researchers focus their attention only on the dispersion of Cu, Ag, Al_2_O_3_ nanoparticles within the base fluid. In this study we developed mathematical model for the squeezing flow analysis of high rate of heating or cooling of base fluids using single and multi-wall carbon nanotubes. Convective boundary conditions are used to sort out heat transfer characteristics. Convergent series solutions are developed by homotopy analysis method[21–27]. Influences of various pertinent parameters on the velocity and temperature distributions are analyzed graphically. Skin friction coefficient and Nusselt number corresponding to different involved parameters are discussed.

## Mathematical Formulation

We consider unsteady squeezing flow of nanofluid between two parallel plates. The lower plate of the channel is fixed at *y* = 0 while the upper plate is at $y=h(t)=\sqrt{\frac{\upsilon_{f}(1-ct)}{a}}$ (which is squeezing towards the lower plate). Heat transfer characteristics are explored with viscous dissipation and convective boundary conditions. Two types of carbon nanotubes (single-wall and multi-wall carbon nanotubes) are used as nanoparticles. Water is chosen as a base fluid. Cartesian coordinates are selected in such a way that *x*-axis is along the flow direction while *y*-axis is normal to it. According to these assumptions the conservation laws take the following forms [15]:

$$\frac{\partial u}{\partial x}+\frac{\partial v}{\partial y}=0,$$ (1)

$$\rho_{nf}\left(\frac{\partial u}{\partial t}+u\frac{\partial u}{\partial x}+v\frac{\partial u}{\partial y}\right)=-\frac{\partial p}{\partial x}+\mu_{nf}\left(\frac{\partial^{2}u}{\partial x^{2}}+\frac{\partial^{2}u}{\partial y^{2}}\right),$$ (2)

$$\rho_{nf}\left(\frac{\partial v}{\partial t}+u\frac{\partial v}{\partial x}+v\frac{\partial v}{\partial y}\right)=-\frac{\partial p}{\partial y}+\mu_{nf}\left(\frac{\partial^{2}v}{\partial x^{2}}+\frac{\partial^{2}v}{\partial y^{2}}\right),$$ (3)

$$\frac{\partial T}{\partial t}+u\frac{\partial T}{\partial x}+v\frac{\partial T}{\partial y}=\frac{k_{nf}}{{\left(\rho c_{p}\right)}_{nf}}\left(\frac{\partial^{2}T}{\partial x^{2}}+\frac{\partial^{2}T}{\partial y^{2}}\right)+\frac{\mu_{nf}}{{\left(\rho c_{p}\right)}_{nf}}\left(4{\left(\frac{\partial u}{\partial x}\right)}^{2}+{\left(\frac{\partial v}{\partial x}+\frac{\partial u}{\partial y}\right)}^{2}\right).$$ (4)

The subjected boundary conditions are

$$\begin{array}{c} u=0,\;v=0,\;-k_{nf}\frac{\partial T}{\partial y}=\gamma_{0}\left(T_{f}-T\right)\;,\;\text{at}\;y=0,\\ u=0,\;v=v_{h}=\frac{dh}{dt},\;T=T_{h},\;\text{as}\;y=h\left(t\right). \end{array}$$ (5)

In the above expressions *u* and *v* denote the velocity components in the x and y-direction respectively, *p* is the pressure, *μ*_*nf*_ is the dynamic viscosity of nanofluids, *ρ*_*nf*_ is the density of nanofluids, (*c*_*p*_)_*nf*_ is the specific heat of nanofluid, *k*_*nf*_ is the thermal conductivity of nanofluids, *v*_*h*_ is the velocity of the upper plate, *T* is the temperature of the fluid, *T*_*f*_ is the temperature of the heated fluid, *h* is the distance between the plates, *γ*_0_ is the heat transfer coefficient and *T*_*h*_ is the temperature of the squeezing plate or upper plate.

Xue [28] analyzed that proposed nanofluid model is valid only for spherical or rotational elliptical particles with small axial ratio. These models do not describe the properties of space distribution of the CNTs on thermal conductivity. To fill this void, Xue [28] proposed a theoretical model based on Maxwell theory considering rotational elliptical nanotubes with very large axial ratio and compensating the effects of space distribution on CNTs.

The values appeared in Eqs (2) and (3) are

$$\begin{array}{l} \mu_{nf}=\frac{\mu_{f}}{{\left(1-\phi \right)}^{2.5}},\;\nu_{nf}=\frac{\mu_{nf}}{\rho_{nf}},\;\rho_{nf}=\left(1-\phi \right)\;\rho_{f}+\phi \rho_{CNT},\\ \alpha_{nf}=\frac{k_{nf}}{\rho_{nf}{\left(c_{p}\right)}_{nf}},\;\frac{k_{nf}}{k_{f}}=\frac{\left(1-\phi \right)+2\phi \frac{k_{CNT}}{k_{CNT}-k_{f}}\text{ln}\frac{k_{CNT}+k_{f}}{2k_{f}}}{\left(1-\phi \right)+2\phi \frac{kf}{k_{CNT}-k_{f}}\text{ln}\frac{k_{CNT}+k_{f}}{2k_{f}}}, \end{array}$$ (6)

where *ϕ* is the nanoparticle volume fraction, *α*_*nf*_ is the thermal diffusivity, *ρ*_*f*_ is the density of the fluid, *k*_*f*_ and *k*_*nf*_ are the thermal conductivities of fluid and nanofluids respectively while *k*_*CNT*_ is the thermal conductivity of carbon nanotubes.

Transformations are taken as follows:

η=yH1−ct,u=(ax2(1−ct))f′(η),H=υfav=−aH21−ctf(η),θ(η)=T−ThTf−Th. (7)

Incompressibility condition (1) is satisfied automatically and after eliminating pressure gradient from Eqs (2) and (3) and then applying these transformations the law of conservation of momentum and energy are reduced as follow:

$$\left(\frac{1}{{}^{{\left(1-\phi \right)}^{2.5}\left(1-\phi +\phi \frac{\rho_{CNT}}{\rho_{f}}\right)}}\right)\;f^{\prime \prime \prime \prime }-\frac{Sq}{2}(3f^{\prime \prime }+\eta f^{\prime \prime \prime })-f^{{}^{\prime }}f^{\prime \prime }+ff^{\prime \prime \prime }=0,$$ (8)

$$\left(\frac{k_{nf}/k_{f}}{\left(1-\phi +\phi \frac{{\left(\rho c_{p}\right)}_{CNT}}{{\left(\rho c_{p}\right)}_{f}}\right)}\right)\;\theta^{\prime \prime }+\text{Pr}\;Sq\left(f\theta^{\prime }-\eta \theta^{\prime }\right)+\frac{\text{Pr}\;Ec}{{\left(1-\phi \right)}^{2.5}}\left(f^{\prime \prime 2}+4\delta^{2}f^{\prime 2}\right)=0,$$ (9)

with the boundary conditions

$$\begin{array}{l} f(0)=0,\;f^{\prime }(0)=0,\;\theta^{\prime }\left(0\right)=-\frac{\beta }{\frac{k_{nf}}{k_{f}}}(1-\theta \left(0\right)),\\ \;f(1)=1,\;f^{\prime }\left(1\right)=0\;\theta \left(1\right)=0 \end{array}$$ (10)

where *Sq* is the squeezing parameter, *Ec* is the Eckert number, Pr is the Prandtl number, *β* is the Biot number and *δ* is the length parameter. These parameters are

$$\begin{array}{l} Sq=\frac{c}{a},\;\text{Pr}=\frac{\mu_{f}c_{p}}{k},\;\beta =\frac{\gamma_{0}H\sqrt{1-ct}}{k_{f}},\\ Ec=\frac{a^{2}x^{2}}{4c_{p}{\left(1-at\right)}^{2}\left(T_{h}-T_{f}\right)},\;\delta =\frac{H\sqrt{1-ct}}{x}. \end{array}$$ (11)

Skin friction coefficient and local Nusselt number are given by

$$\begin{array}{l} \tau_{w}=\mu_{nf}{\left(\frac{\partial u}{\partial y}\right)}_{y=0},\;q_{w}=-\kappa_{nf}{\left(\frac{\partial T}{\partial y}\right)}_{y=0},\\ C_{f}=\frac{\tau_{w}}{\rho_{f}U_{w}^{2}},\;Nu_{x}=\frac{xq_{w}}{k_{f}(T_{f}-T_{h})}. \end{array}$$ (12)

Dimensionless skin friction coefficient and local Nusselt number are

$$C_{f}{\text{Re}}_{x}^{1/2}=\frac{1}{{\left(1-\phi \right)}^{2.5}}f^{\prime \prime }(0),\;Nu_{x}{\text{Re}}_{x}^{-1/2}=-\frac{k_{nf}}{k_{f}}\theta^{\prime }(0),$$ (13)

where Re_*x*_ = *v*_*h*_*x* / *ν*_*f*_ is the local Reynolds number.

## Homotopic Solutions

Homotopy analysis method (HAM) is proposed by Liao [22] in 1992, used to find the solution of nonlinear differential equations. Homotopy analysis method has several advantages such as (i) it is independent of small or large values of the parameters. (ii) It guarantees the convergence of the solution and (iii) it provides a great freedom for the selection of base function and linear operator.

As homotopy analysis method gives the series solution of any differential equation, therefore it requires the initial approximations to proceed the series solutions. Here initial approximations satisfying the imposed conditions in the problems are

$$\begin{array}{c} f_{0}\left(\eta \right)=A_{1+}A_{2}\eta +A_{3}\eta^{2}+A_{4}\eta^{3},\;\theta_{0}\left(\eta \right)=A_{5}+A_{6}\eta ,\\ L_{f}\left(f\right)=\frac{d^{4}f}{d\eta^{4}},\;L_{\theta }\left(\theta \right)=\frac{d^{2}\theta }{d\eta^{2}}, \end{array}$$ (14)

with

$$\begin{array}{l} L_{f}\left[A_{1+}A_{2}\eta +A_{3}\eta^{2}+A_{4}\eta^{3}\right]=0,\\ L_{\theta }\left[A_{5}+A_{6}\eta \right]=0, \end{array}$$ (15)

where *A*_*i*_(*i* = 1,2,…,6) are the arbitrary constants. The zeroth and *m*th order deformation problems are:

### Zeroth-Order Problem

$$\begin{array}{l} \left(1-p\right)L_{f}\left[\overset{⌢}{f}\left(\eta ;p\right)-f_{0}\left(\eta \right)\right]=p\hbar_{f}N_{f}\left[\overset{⌢}{f}\left(\eta ;p\right),\overset{⌢}{\theta }\left(\eta ;p\right)\right],\\ \left(1-p\right)L_{\theta }\left[\overset{⌢}{\theta }\left(\eta ;p\right)-\theta_{0}\left(\eta \right)\right]=p\hbar_{\theta }N_{\theta }\left[\overset{⌢}{\theta }\left(\eta ;p\right),\overset{⌢}{f}\left(\eta ;p\right)\right],\\ \overset{⌢}{f}\left(0;p\right)=0,\;{\overset{⌢}{f}}^{\prime }\left(0;p\right)=0,\;\overset{⌢}{f}\left(1;p\right)=1,\;{\overset{⌢}{f}}^{\prime }\left(1;p\right)=0, \end{array}$$ (16)

$${\overset{⌢}{\theta }}^{\prime }\left(0;p\right)=-\frac{\beta }{\frac{k_{nf}}{k_{f}}}\left(1-\overset{⌢}{\theta }\left(0;p\right)\right),\;\overset{⌢}{\theta }\left(1;p\right)=0,$$ (17)

$$\begin{array}{l} N_{f}\left[\overset{⌢}{f}\left(\eta ,p\right),\overset{⌢}{\theta }\left(\eta ;p\right)\right]=\left(\frac{1}{{}^{{\left(1-\phi \right)}^{2.5}\left(1-\phi +\phi \frac{\rho_{CNT}}{\rho_{f}}\right)}}\right)\frac{\partial^{4}\overset{⌢}{f}\left(\eta ;p\right)}{\partial \eta^{4}}-\\ Sq\left(\eta \frac{\partial^{3}\overset{⌢}{f}\left(\eta ;p\right)}{\partial \eta^{3}}+3\frac{\partial^{2}\overset{⌢}{f}\left(\eta ;p\right)}{\partial \eta^{2}}+\frac{\partial \overset{⌢}{f}\left(\eta ;p\right)}{\partial \eta }\frac{\partial^{2}\overset{⌢}{f}\left(\eta ;p\right)}{\partial \eta^{2}}-\overset{⌢}{f}\left(\eta ;p\right)\frac{\partial^{3}\overset{⌢}{f}\left(\eta ;p\right)}{\partial \eta^{3}}\right), \end{array}$$ (18)

$$\begin{array}{l} N_{\theta }\left[\overset{⌢}{\theta }\left(\eta ;p\right),\overset{⌢}{f}\left(\eta ;p\right)\right]=\left(\frac{k_{nf}/k_{f}}{\left(1-\phi +\phi \frac{{\left(\rho c_{p}\right)}_{CNT}}{{\left(\rho c_{p}\right)}_{f}}\right)}\right)\frac{\partial^{2}\overset{⌢}{\theta }(\eta ,p)}{\partial \eta^{2}}+\text{Pr}\;Sq\left(\;\overset{⌢}{f}\left(\eta ;p\right)\frac{\partial \overset{⌢}{\theta }(\eta ,p)}{\partial \eta }-\eta \frac{\partial \overset{⌢}{\theta }(\eta ,p)}{\partial \eta }\right)\\ +\frac{\text{Pr}\;Ec}{{\left(1-\phi \right)}^{2.5}}\left({\left(\frac{\partial^{2}\overset{⌢}{\theta }(\eta ,p)}{\partial \eta^{2}}\right)}^{2}+4\delta^{2}{\left(\frac{\partial \overset{⌢}{f}\left(\eta ;p\right)}{\partial \eta }\right)}^{2}\right), \end{array}$$ (19)

in which *p* ∈ [0,1] is embedding parameter and ℏ_*f*_, ℏ_*θ*_ are the non-zero auxiliary parameters.

### *m*th-Order Deformation Problems

Here

$$\begin{array}{l} L_{f}\left[f_{m}\left(\eta \right)-\chi_{m}f_{m-1}\left(\eta \right)\right]=\hbar_{f}R_{m}^{f}\left(\eta \right),\\ L_{\theta }\left[\theta_{m}\left(\eta \right)-\chi_{m}\theta_{m-1}\left(\eta \right)\right]=\hbar_{\theta }R_{m}^{\theta }\left(\eta \right), \end{array}$$ (20)

$$\begin{array}{l} f_{m}\left(0\right)=0,\;f_{m}^{\prime }\left(0\right)=0,\;f_{m}\left(1\right)=0,\;f_{m}^{\prime }\left(1\right)=0,\\ \theta_{m}^{\prime }\left(0\right)=\frac{\beta }{\frac{k_{nf}}{k_{f}}}\theta_{m}\left(0\right),\;\theta_{m}\left(1\right)=0, \end{array}$$ (21)

$$R_{m}^{f}\left(\eta \right)=\left(\frac{1}{{}^{{\left(1-\phi \right)}^{2.5}\left(1-\phi +\phi \frac{\rho_{CNT}}{\rho_{f}}\right)}}\right)f_{m-1}^{\prime \prime \prime \prime }-\frac{Sq}{2}(3f_{m-1}^{\prime \prime }+\eta f_{m-1}^{\prime \prime \prime })+\overset{m-1}{\underset{k=0}{\sum }}\left(f_{m-1-k}^{{}^{\prime }}f_{k}^{\prime \prime }-f_{m-1-k}f_{k}^{\prime \prime \prime }\right),$$ (22)

$$\begin{array}{c} R_{m}^{\theta }\left(\eta \right)=\left(\frac{k_{nf}/k_{f}}{\left(1-\phi +\phi \frac{{\left(\rho c_{p}\right)}_{CNT}}{{\left(\rho c_{p}\right)}_{f}}\right)}\right)\theta_{m-1}^{\prime \prime }+\text{Pr}\;Sq\left(\sum_{k=0}^{m-1}f_{m-1-k}\theta_{k}^{\prime }-\eta \theta_{m-1}^{\prime }\right)\\ +\frac{\text{Pr}\;Ec}{{\left(1-\varphi \right)}^{2.5}}\sum_{k=0}^{m-1}\left(f_{m-1-k}^{\prime \prime }f_{k}^{\prime \prime }+4\delta^{2}f_{m-1-k}^{\prime }f_{k}^{\prime }\right), \end{array}$$ (23)

$$\chi_{m}=\{\begin{array}{c} 0,\;m\leq 1\\ 1,\;m>1 \end{array}.$$ (24)

For *p* = 0 and *p* = 1, we can write

$$\begin{array}{l} \overset{⌢}{f}\left(\eta ;0\right)=f_{0}\left(\eta \right),\;\overset{⌢}{f}\left(\eta ;1\right)=f\left(\eta \right),\\ \overset{⌢}{\theta }\left(\eta ;0\right)=\theta_{0}\left(\eta \right),\;\overset{⌢}{\theta }\left(\eta ;1\right)=\theta \left(\eta \right), \end{array}$$ (25)

and with the variation of *p* from 0 to 1, $\overset{⌢}{f}\left(\eta ;p\right)$ and $\overset{⌢}{\theta }\left(\eta ;p\right)$ vary from the initial solutions *f*_0_(*η*) and *θ*_0_(*η*) to the final solutions *f*(*η*) and *θ*(*η*) respectively. By Taylor series we have

$$\begin{array}{l} \overset{⌢}{f}\left(\eta ;p\right)=f_{0}\left(\eta \right)+\sum_{m=1}^{\infty }f_{m}\left(\eta \right)\;p^{m},\;f_{m}\left(\eta \right)={\frac{1}{m!}\frac{\partial^{m}\overset{⌢}{f}\left(\eta ;p\right)}{\partial p^{m}}|}_{p=0},\\ \overset{⌢}{\theta }\left(\eta ;p\right)=\theta_{0}\left(\eta \right)+\sum_{m=1}^{\infty }\theta_{m}\left(\eta \right)\;p^{m},\;\theta_{m}\left(\eta \right)={\frac{1}{m!}\frac{\partial^{m}\overset{⌢}{\theta }\left(\eta ;p\right)}{\partial p^{m}}|}_{p=0}. \end{array}$$ (26)

The value of auxiliary parameter is chosen in such a way that the series (32) and (33) converge at *p* = 1 i.e.

$$\begin{array}{l} f\left(\eta \right)=f_{0}\left(\eta \right)+\sum_{m=1}^{\infty }f_{m}\left(\eta \right),\\ \theta \left(\eta \right)=\theta_{0}\left(\eta \right)+\sum_{m=1}^{\infty }\theta_{m}\left(\eta \right). \end{array}$$ (27)

The general solutions (*f*_*m*_, *θ*_*m*_) of Eqs (24) and (25) in terms of special solutions $\left(f_{m}^{*},\;\theta_{m}^{*}\right)$ are given by

$$\begin{array}{l} f_{m}\left(\eta \right)=f_{m}^{?}\left(\eta \right)+A_{1+}A_{2}\eta +A_{3}\eta^{2}+A_{4}\eta^{3},\\ \theta_{m}\left(\eta \right)=\theta_{m}^{?}\left(\eta \right)+A_{5}+A_{6}\eta , \end{array}$$ (28)

### Convergence Analysis

**Fig 1:**
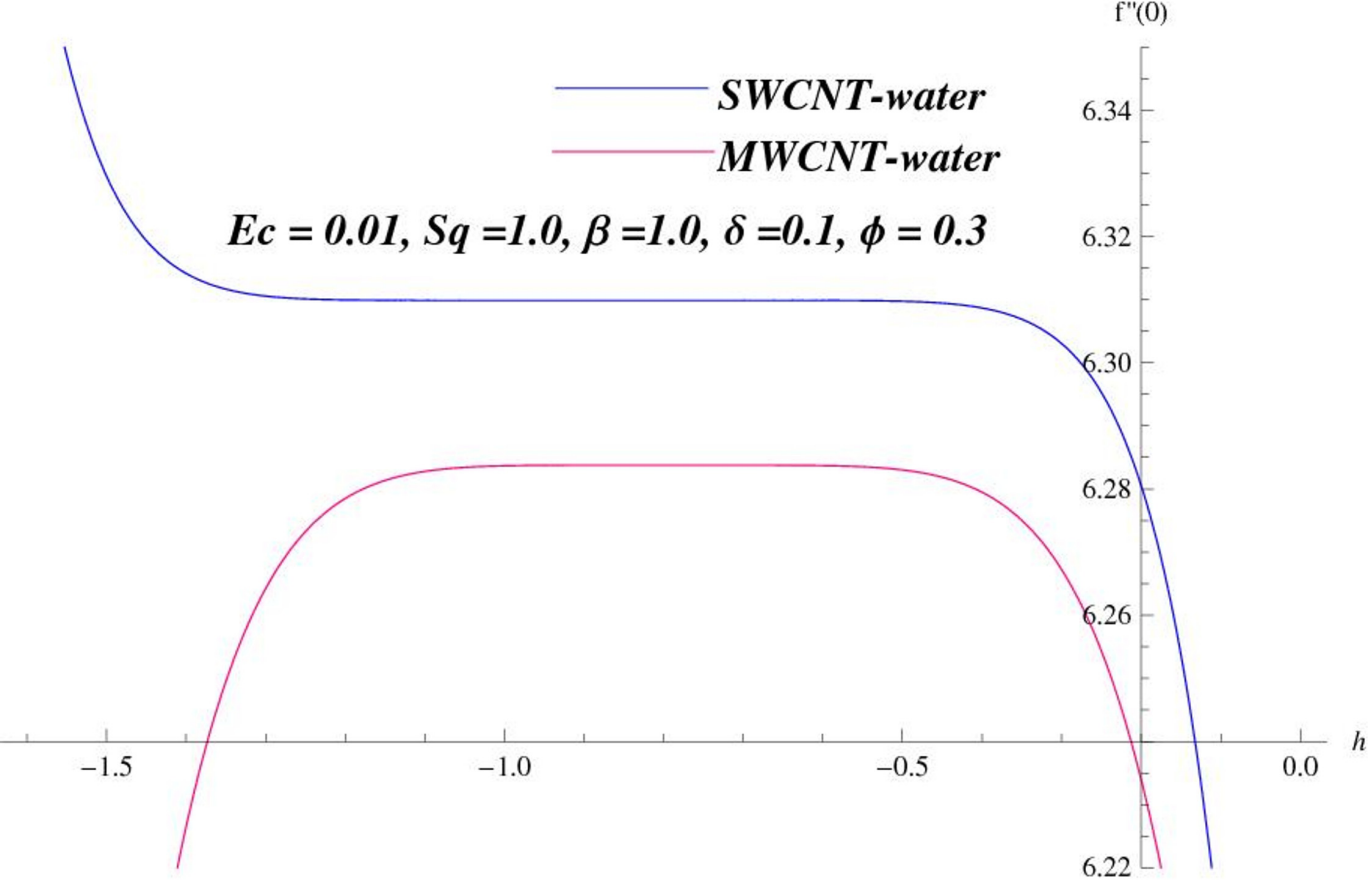
ℏ–curves for *f*.

**Fig 2:**
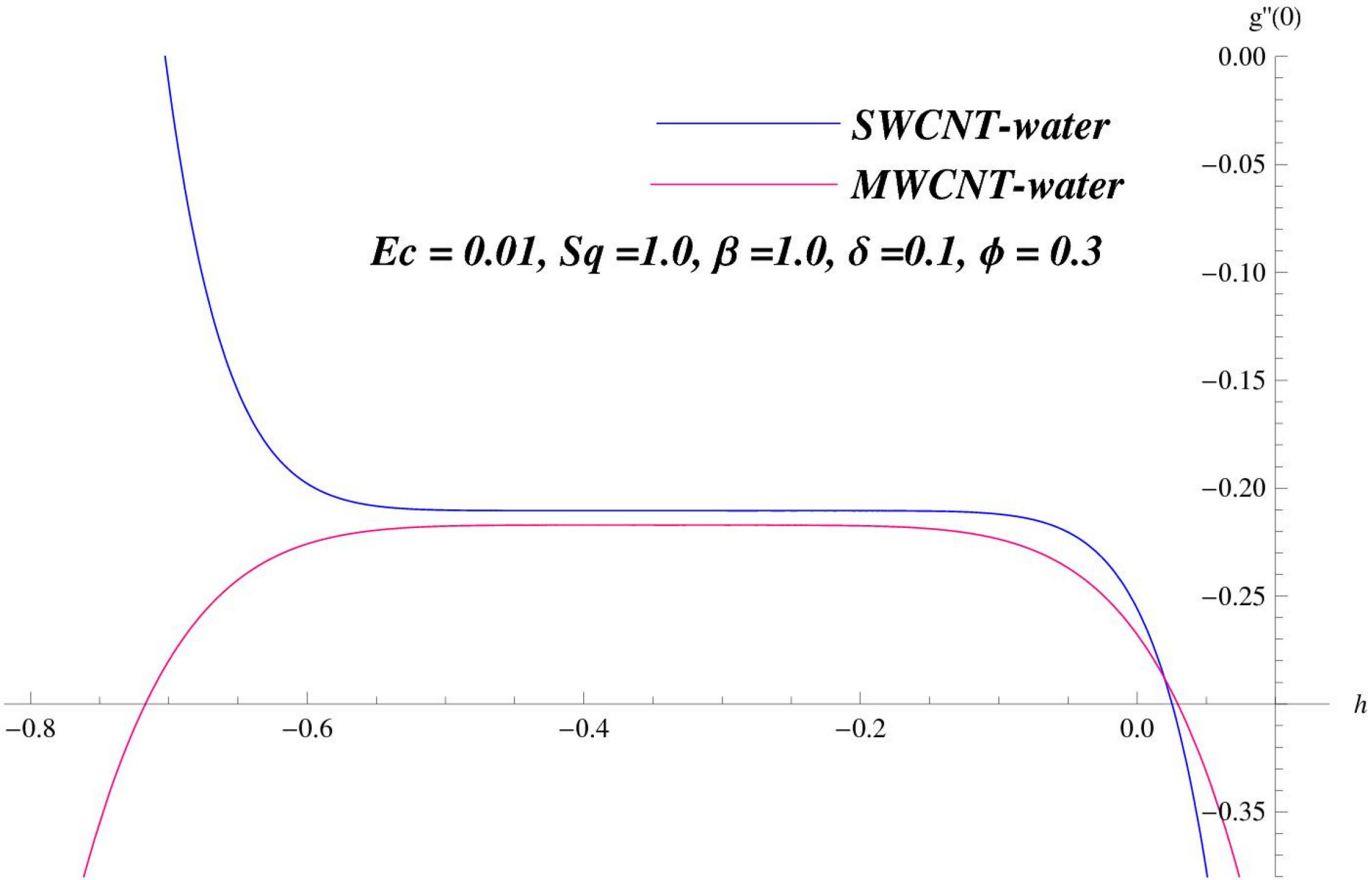
ℏ–curves for *θ*.

Homotopy analysis method was first proposed by Liao [21] in 1992 which is used to obtain the solutions of highly nonlinear problems. The h-curves in Figs 1 and 2 are displayed for convergence region. The admissible ranges of the auxiliary parameters ℏ_*f*_ and ℏ_*θ*_ for SWCNT case are −1.26 ≤ ℏ_*f*_ ≤ −0.5 and −0.56 ≤ ℏ_*θ*_ ≤ −0.1 while for MWCNT case these values are −1.3 ≤ ℏ_*f*_ ≤ −0.52 and −0.57 ≤ ℏ_*θ*_ ≤ −0.18.

## Discussion

**Fig 3:**
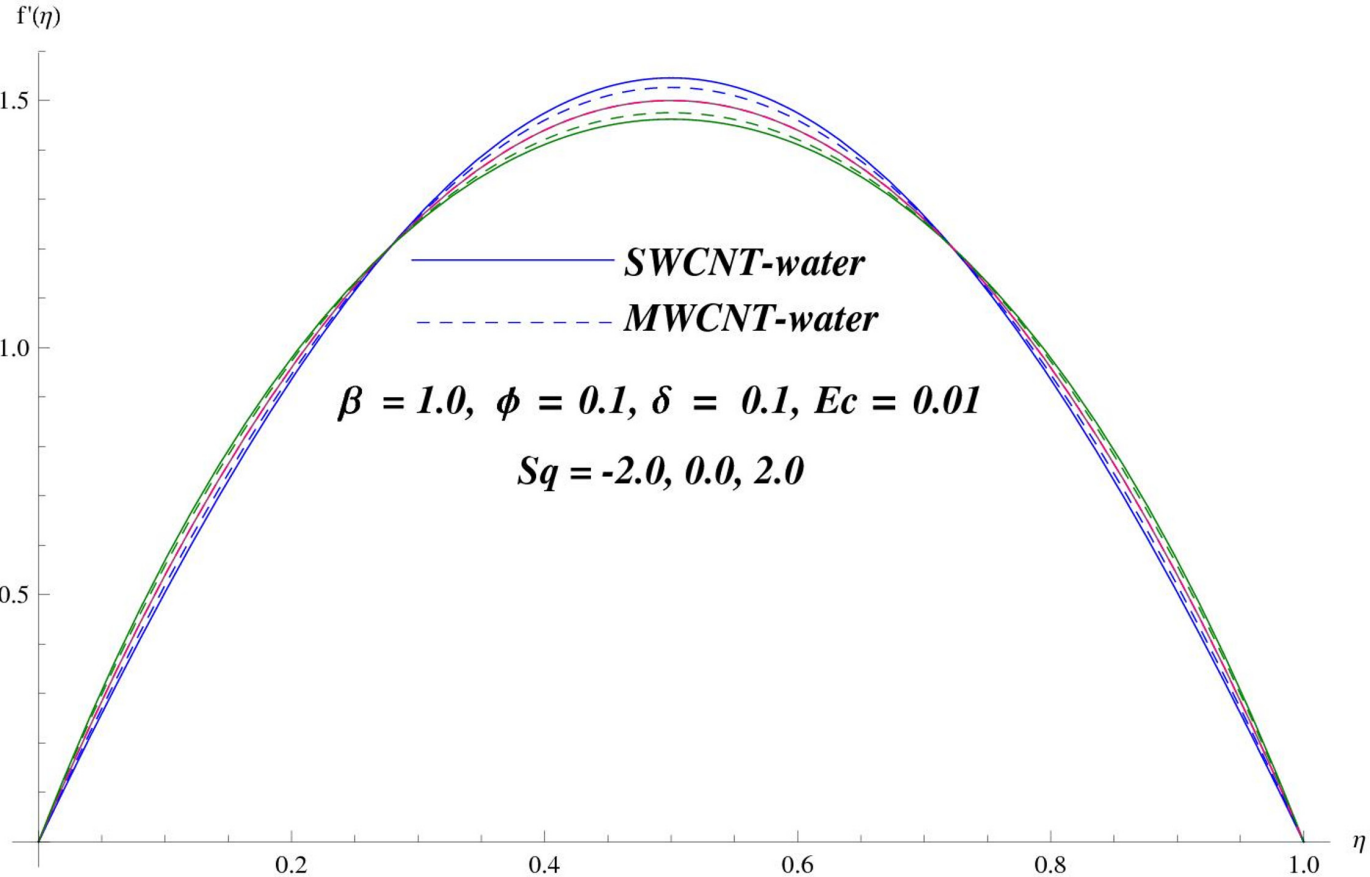
Effect of *Sq* on *f* ^'^.

**Fig 4:**
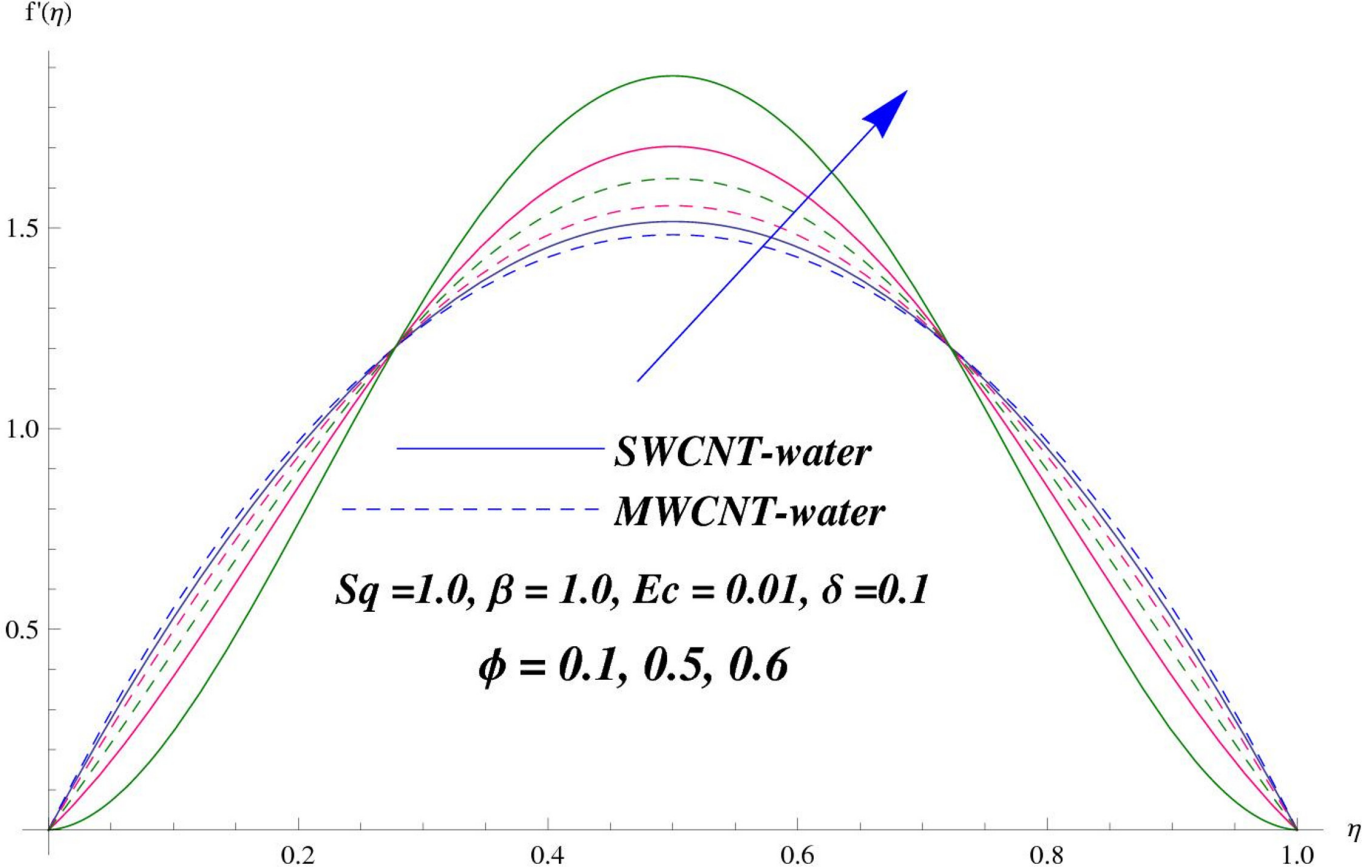
Effect of *ϕ* on *f* ^'^.

**Fig 5:**
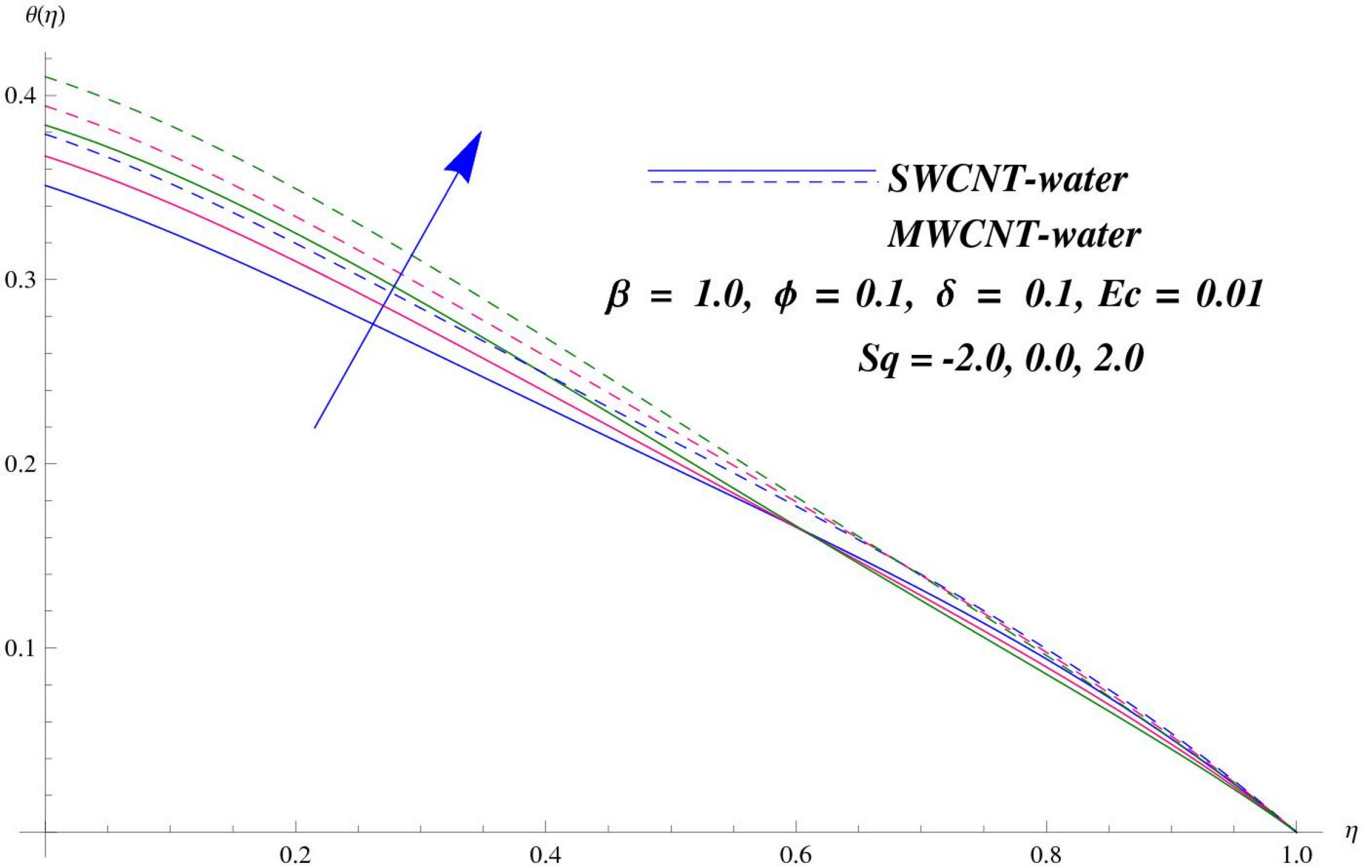
Effect of *Sq* on *θ*.

**Fig 6:**
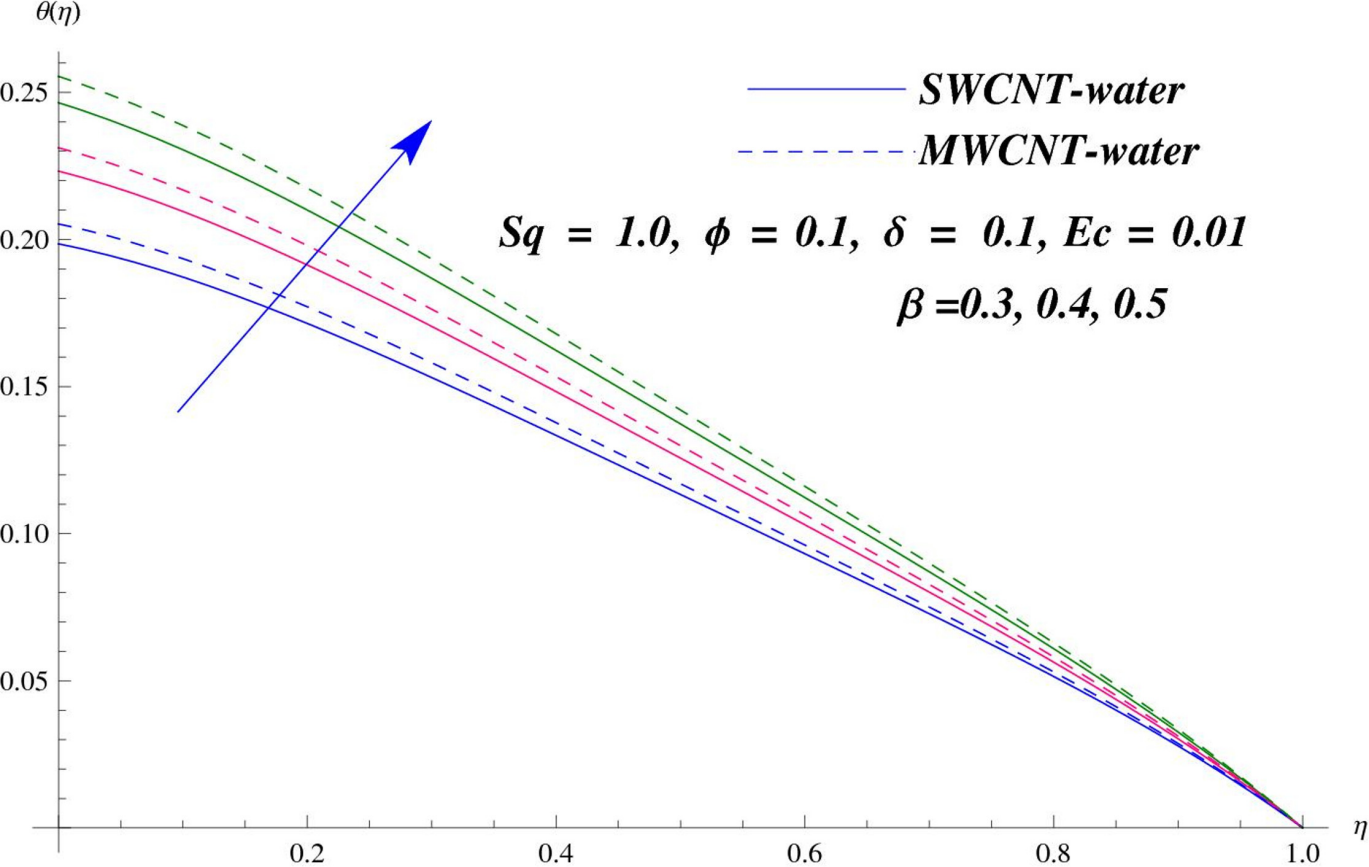
Effect of *β* on *θ*.

**Fig 7:**
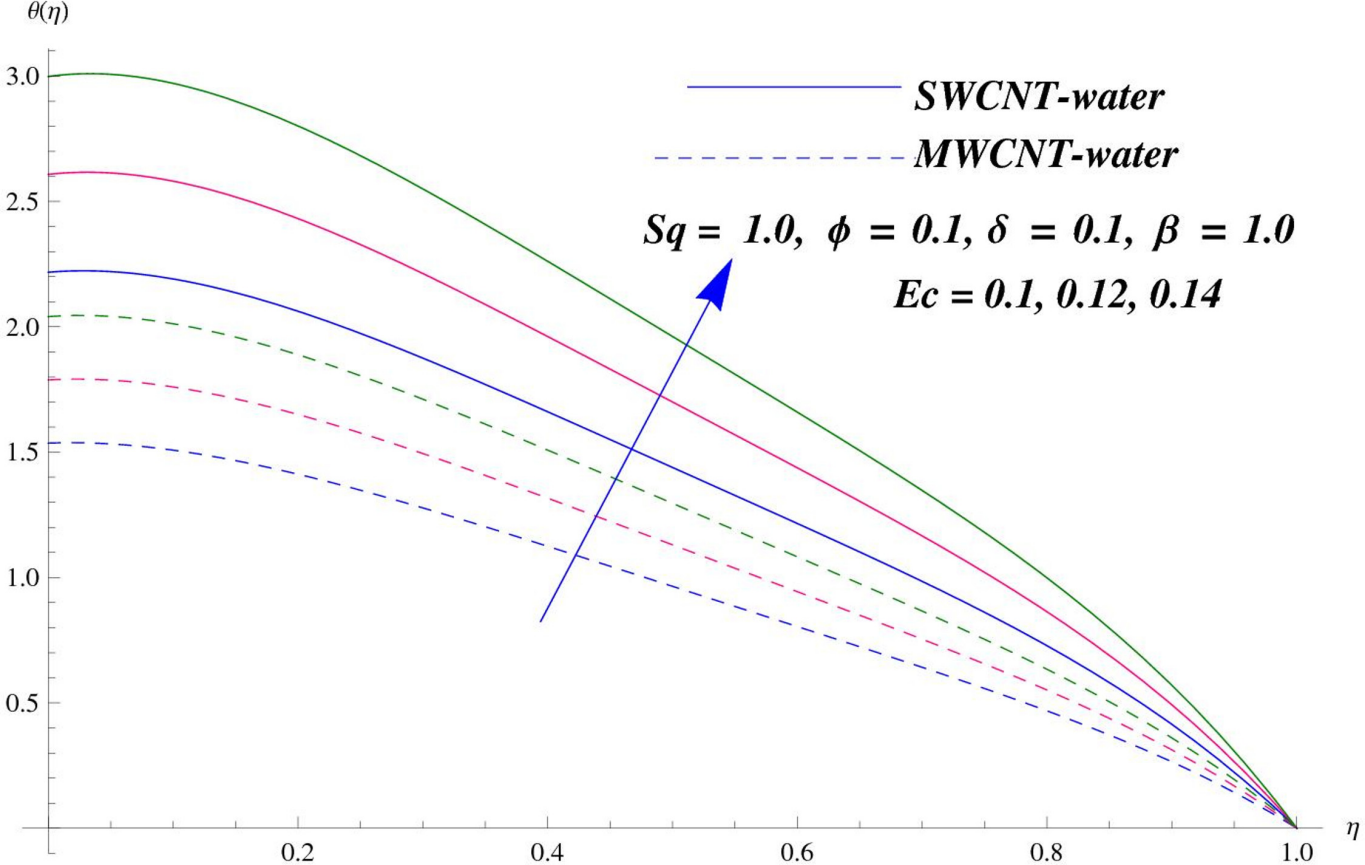
Effect of *E*_*C*_ on *θ*.

**Fig 8:**
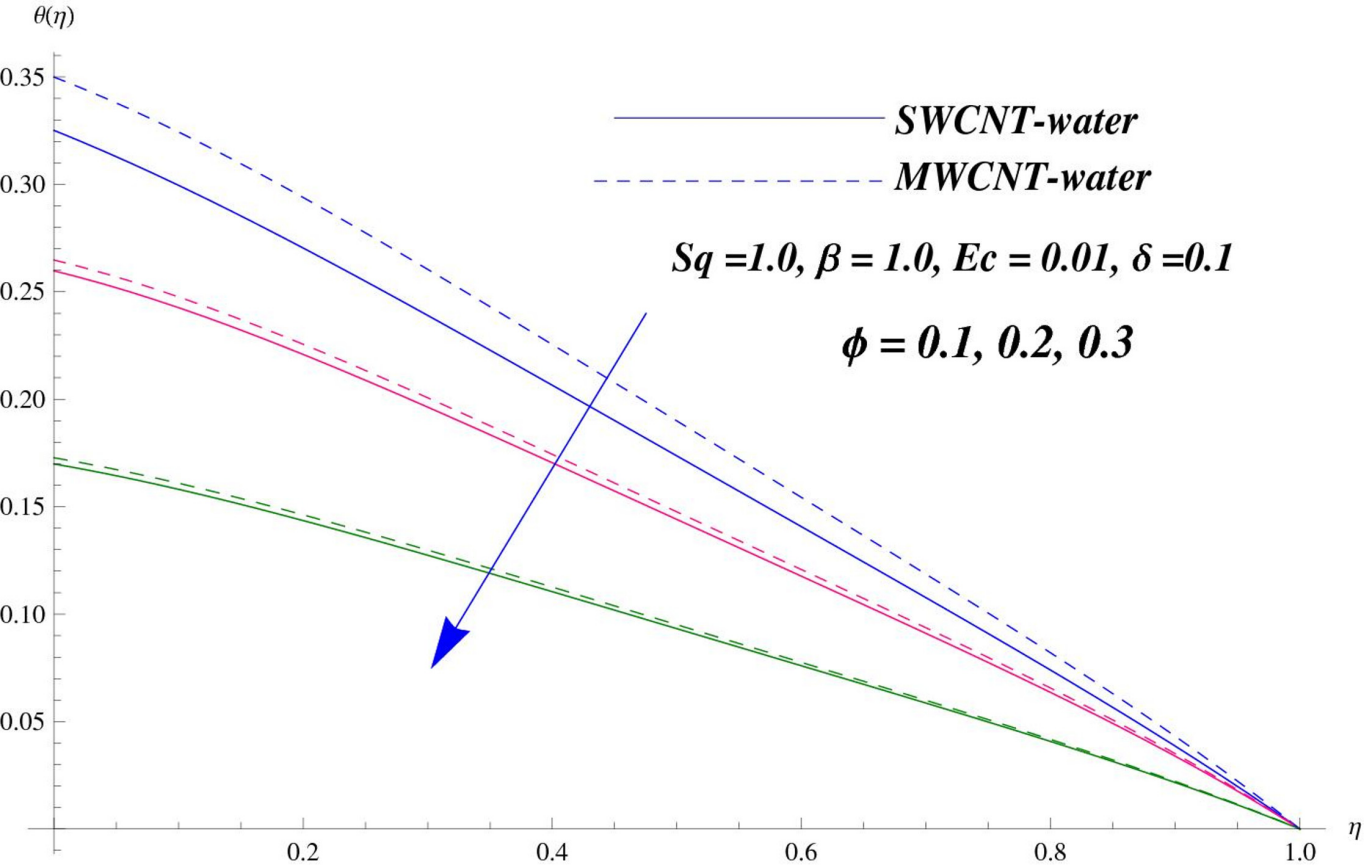
Effect of *ϕ* on *θ*.

**Fig 9:**
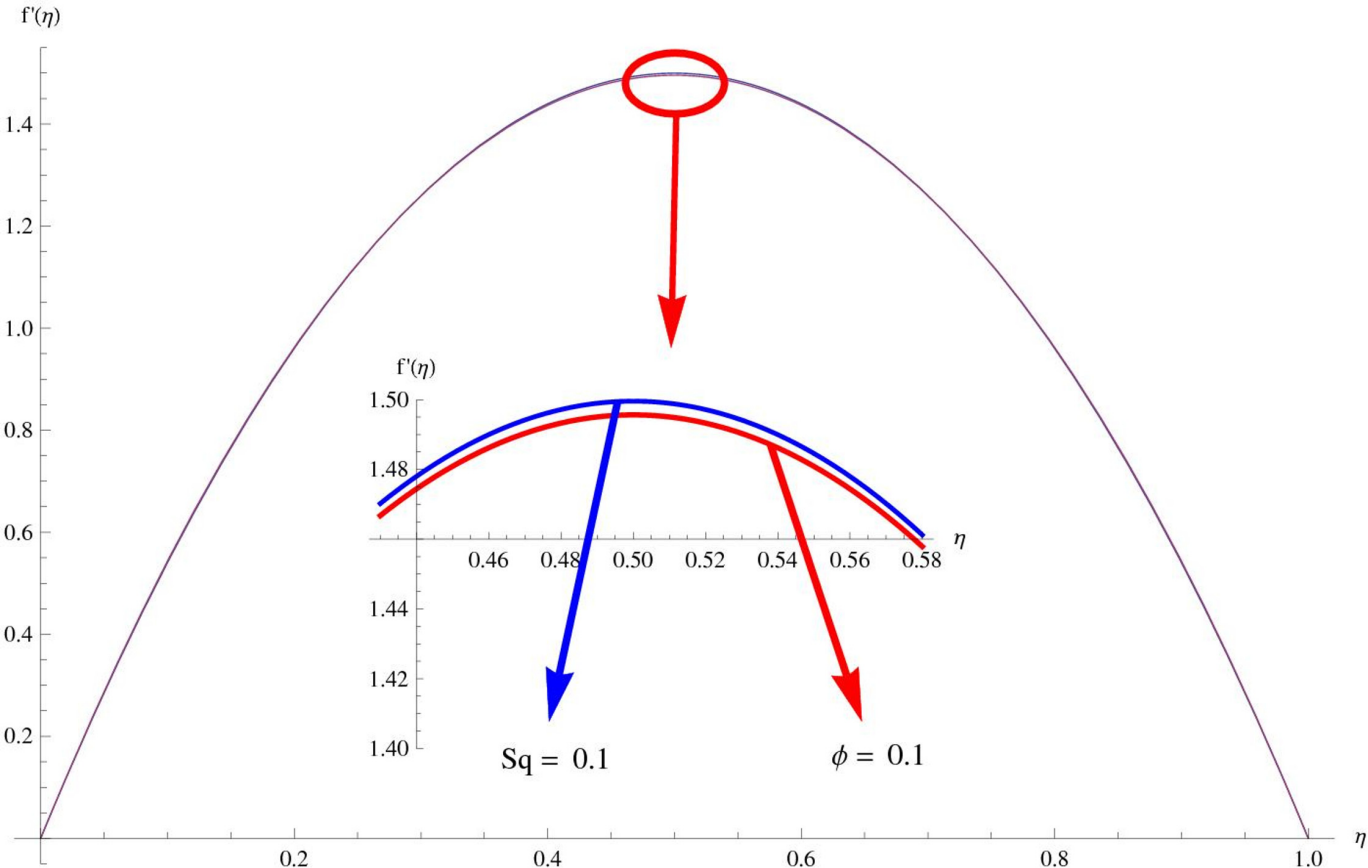
Comparison of effects of *Sq* and *ϕ* on *f* ^'^.

**Fig 10:**
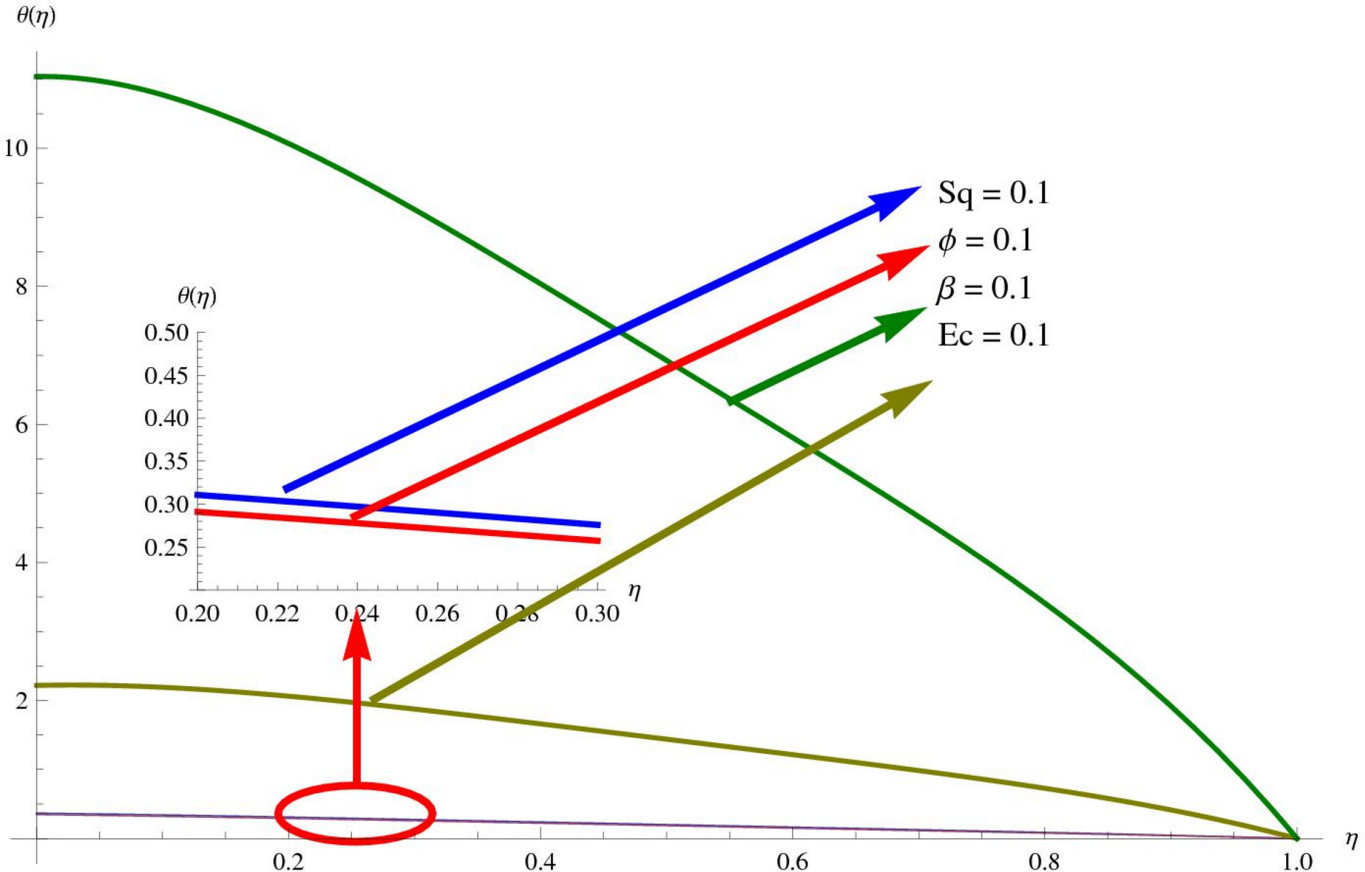
Comparison of Effects of *Sq*, *ϕ*, *β* and *E*_*C*_ on *θ*.

The main purpose of this section is to elaborate the physical behavior of various pertinent parameters on the velocity and temperature distributions corresponding to single and multi-wall carbon nanotubes. Fig 3 shows the effect of squeezing parameter *Sq* on the velocity profile. Here *Sq* < 0 corresponds to the motion of upper plate away from the lower plate. It provides more space between the plates in which fluid to be imprisoned. To fill this space, the fluid moves in reverse direction and thus velocity profile decreases. Similarly *Sq* > 0 corresponds to the motion of the upper plate towards the lower plate, due to which a force (squeezing force) is experienced by the fluid which is responsible for the motion of the fluid with more velocity. As a result, velocity profiles enhances. Further the velocity profile is dominant for the multi-wall carbon nanotubes when compared with single-wall carbon nanotubes. Behavior of nanoparticle volume fraction *ϕ* on the velocity distribution is portrayed in Fig 4. It is observed that the velocity profile reduces for larger values of nanoparticle volume fraction near the plates while it enhances away from the plates for both SWCNTs and MWCNTs cases. Also the effect of MWCNTs dominants over SWCNTs due to the low density. Impact of squeezing parameter *Sq* on the temperature profile is depicted in Fig 5. It is analyzed that squeezing parameter *Sq* < 0. i.e., when squeezing plate moves away from the lower plate then there is reduction of temperature profile while opposite behavior is observed for *Sq* > 0, i.e., when the squeezing plate moves towards the lower plate. Further MWCNTs show dominant behavior when compared to SWCNTs. Analysis of Biot number *β* on the temperature profile is sketched in Fig 6 for both SWCNTs and MWCNTs cases. Temperature profile shows increasing behavior for larger values of *β*. In fact, when we increase Biot number, the heat transfer rate increases which is responsible for rise in temperature of the fluid. Behavior of Eckert number *Ec* on the temperature profile is displayed in Fig 7. It is noted that temperature profile is higher for larger values of Eckert number. In fact, larger Eckert number corresponds to higher drag forces between the fluid particles. As a result, more heat is produced and thus temperature profile increases. Analysis of nanoparticle volume fraction *ϕ* on temperature distribution is illustrated in Fig 8 for SWCNTs and MWCNTs. Temperature distribution shows decreasing behavior for larger nanoparticle volume fraction. Single-wall carbon nanotubes show dominant behavior on the temperature distribution than the multi-wall carbon nanotubes. Fig 9 is sketched to compare the effects of squeezing parameter *Sq* and nanoparticle volume fraction *ϕ* on the velocity profile. It is observed that the effect of *Sq* is more than dominant *ϕ* on the velocity of the fluid. Fig 10 is portrayed for comparison of effects of *ϕ*, *Sq*, *Ec* and *β* on the temperature profile. It is found out that the effect of *β* is maximum on the temperature profile which is followed by *Ec*, *Sq* and *ϕ*.

Table 1 demonstrates the thermophysical properties (i.e., density, specific heat and thermal conductivity) of the base fluid water and carbon nanotubes. Table 2 displays the convergence of series solutions for the conservation laws of momentum and energy. It is noted that 67^th^ and 60^th^ order of approximations are sufficient for the convergence of momentum and energy equations in the case of SWCNTs respectively. However for MWCNTs the 67^th^ and 50^th^ order of approximations are sufficient for the convergence of momentum and energy equations. Table 3 is prepared for the numerical values of skin friction coefficient for different values of various pertinent parameters. It is observed that the skin friction coefficient is higher for larger values of squeezing parameter *Sq*, nanoparticle volume fraction *f* and Biot number *β* while it decreases for larger Eckert number for both single-wall and multi-wall carbon nanotubes. Table 4 is presented for the numerical values of Nusselt number under the influence of various involved parameters. It is found out that the rate of heat transfer reduces for larger values of squeezing parameter *Sq* and Eckert number *Ec* in both single-wall and multi-wall carbon nanotubes (SWCNTs and MWCNTs) while the rate of heat transfer increases for larger values of nanoparticle volume fraction *ϕ* and Biot number *β* in both SWCNTs and MWCNTs cases.

**Table 1:** Numerical values of thermophysical properties of base fluid and nanoparticles [13] (i.e. density, specific heat and thermal conductivity).

Physical Porperties	Base fluid	Nanoparticles	
	Water	SWCNTs	MWCNTs
*ρ*(*kg* / *m*^3^)	997	2600	1600
*c*_*p*_(*J* / *kgK*)	4179	425	796
*k*(*W* / *mK*)	0.613	6600	3000

**Table 2:** Convergence of series solutions for different order of approximations when *δ* = 0.1, *ϕ* = 0.3, *β* = 1.0, *Sq* = 1.0 and *Ec* = 0.01.

	SWCNT		MWCNT	
Order of approximations	f′′(0)	-θ′(0)	f′′(0)	-θ′(0)
1	6.03571	0.241736	6.03571	0.253125
10	6.21878	0.211558	6.20982	0.218798
20	6.28308	0.210468	6.26448	0.217195
30	6.302	0.210431	6.27874	0.217133
40	6.30756	0.210426	6.28246	0.217128
50	6.30921	0.210424	6.28343	0.217126
60	6.30969	0.210423	6.28368	0.217126
67	6.30981	0.210423	6.28374	0.217126
70	6.30981	0.210423	6.28374	0.217126

**Table 3:** Numerical values of skin friction coefficient for different values of various pertinent parameters for both SWCNTs and MWCNTs when *δ* = 0.1.

				SWCNT	MWCNT
Sq	φ	Ec	β	C_f_ $\sqrt{{Re}_{x}}$	C_f_ $\sqrt{{Re}_{x}}$
0.0	0.3	0.01	1.0	14.64	14.64
0.1				14.684	14.675
0.2				14.740	14.719
1.0	0.1	0.01	1.0	8.2113	8.17736
	0.2			10.9425	10.8739
	0.4			22.095	21.9552
1.0	0.3	0.01	0.1	15.1547	15.0509
		0.1		15.1547	15.0509
		0.2		15.1546	15.0507
1.0	0.3	0.01	0.1	15.1546	15.052
			0.3	15.1547	15.0509
			0.5	15.1548	15.051

**Table 4:** Numerical values of Nusselt number for different values of various pertinent parameters for both SWCNTs and MWCNTs when *δ* = 0.1.

				SWCNT	MWCNT
Sq	φ	Ec	β	${Nu}_{x}/\sqrt{{Re}_{x}}$	${Nu}_{x}/\sqrt{{Re}_{x}}$
0.0	0.3	0.01	1.0	0.7958	0.7958
0.1				0.7796	0.7956
0.2				0.7794	0.7955
1.0	0.1	0.01	1.0	0.6120	0.5934
	0.2			0.7344	0.7163
	0.4			0.8252	0.8102
1.0	0.3	0.01	0.1	0.7943	0.7781
		0.1		-0.8618	-0.1715
		0.2		-1.0646	-1.2267
1.0	0.3	0.01	0.1	0.0869	0.08691
			0.3	0.2577	0.2541
			0.5	0.4198	0.4131

### Closing Remarks

In the present analysis we have disclosed the characteristics of unsteady squeezing flow of carbon nanotubes (single-wall and multi-wall carbon nanotubes) in the channel of two infinite parallel plates. The key points are summarized as follows:

➢ Velocity distribution shows decreasing behavior for squeezing parameter *Sq* and increasing behavior for nanoparticle volume fraction *ϕ*. Also velocity dominants in case of multi-wall carbon nanotubes.
➢ Temperature profile is higher for multi-wall carbon nanotubes than the single-wall carbon nanotubes when there is an increase in squeezing parameter *Sq*, nanoparticles volume fraction *ϕ*, Biot number *β* and Eckert number *Ec*.
➢ Higher values of squeezing parameter *Sq* enhance the skin friction coefficient but it is lower for MWCNT than the SWCNT case.
➢ Higher values of nanoparticle volume fraction *ϕ*, Eckert number *Ec* and Biot number *β* result in the enhancement of skin friction coefficient for both SWCNTs and MWCNTs cases. However, the effect of SWCNTs dominants over MWCNTs.
➢ Cooling process or rate of heat transfer can be enhanced by using smaller values of squeezing parameter and Eckert number while it increase for larger values of Biot number *β* and nanoparticle volume fraction *ϕ*. The case of SWCNT is found more efficient.
